# Event and Comment

**Published:** 1911-10-15

**Authors:** 


### DENTAL REGISTER.

**Vol. LXV. October 15, 1911. No. 10**

### EVENT AND COMMENT.

THE COMMERCIAL IN EDUCATION. As this is the season when the Dental Colleges are beginning new sessions one can not help speculating as whether the managers of our schools are making any advances in their efforts to secure a better educated class of students. We have just received a letter from an American dentist who is practicing in a far eastern country, who wrote to several dental colleges in this country for their announcements in behalf of a friend who was interested in dental education. He says some of the letters and inducements to attend their colleges are so unprofessional as to be rediculously amusing, and would pass for smart advertising by a gold brick promoter.

A few years ago we received a paper written by a recent graduate of one of our best schools on the methods of advertising used by our dental colleges. He called attention to the unprofessional statements in the advertisements of the dental colleges in our professional journals. His method was to take a number of prominent college advertisements and convert them into advertisements of their respective Deans for private practice, and the illustration was most effective indeed. For instance Dr. James A. Peterson, A.B., M.D., D.D.S., etc., is the owner of the finest dental office in the world, occupying more than 30,000 cubic feet of floor space, which is equipped with the newest chairs, fountain cuspidors, electric lathes, etc., etc., with twenty-seven of the most eminent and skillful men to be found in the profession as assistants; he is prepared to execute the best kind of dentistry known to the world. Obviously we could not print such a paper. If any one wants to see how absurd and unethical some of these advertisements are, let him take advertisement of almost any school and read the dean's name into it as a private matter and see what he will think of some of our most respected deans.

Undoubtedly most of our schools must be maintained, or their proprietors think they must be, by the usual commercial methods, because prospective dental students being ignorant of ethical practices, will not discriminate in favor of the modest and ethical statments. This probably accounts for the fact that it is so hard to raise entrance standards. We have recruited our profession so many years from the unsophisticated that we have failed to discriminate against the unlettered, and the practice has become habitual rather than intentional. Is it not high time that college managers were beginning to see that they must change their methods, or they can never appeal to the better educated youth nor satisfy the higher ideals of our profession? There are thousands of bright young men graduating from our high schools and colleges every year who would be glad to enter our profession if they could feel that it was a cultured and dignified calling such as most of us believe it to be, and are doing what we can to make it so. If we must appeal to men by the commercial methods, why not make our appeal to the educated classes who will more easily take up with our higher professional ideals, and so dignify our calling? The burden of grounding a well prepared student ought not be so difficult as one of doubtful preparation; and the expense and vexation of conducting a college should not be as great as at present. There would certainly be less opportunity for professional criticism. Here is certainly an opportunity to make a grand step upward, and it not only can, but should be taken.

